# The Vascular Architecture of Macular Neovascularization in Age-Related Macular Degeneration as a Predictor of Therapy Requirements: A 3-Year Longitudinal Analysis

**DOI:** 10.3390/diagnostics15080982

**Published:** 2025-04-12

**Authors:** Michael Grün, Kai Rothaus, Martin Ziegler, Clemens Lange, Albrecht Lommatzsch, Henrik Faatz

**Affiliations:** 1 Department of Ophthalmology at St. Franziskus Hospital, 48145 Münster, Germany; 2 Department of Ophthalmology, Freiburg University Hospital, 79106 Freiburg, Germany; 3 Achim Wessing Institute for Diagnostic Ophthalmology, Duisburg-Essen University, 45147 Essen, Germany

**Keywords:** macular neovascularization, imaging, age-related macular degeneration, OCT angiography, choroidal neovascularization, anti-VEGF therapy, biomarker, prediction

## Abstract

**Background:** Anti-Vascular Endothelial Growth Factor (VEGF) therapy is an effective therapy for improving and stabilizing the vision of patients with neovascular age-related macular degeneration (nAMD). However, the treatment requirements, particularly the number of intraocular injections, can vary significantly among patients. This study aimed to analyze the vascular characteristics of macular neovascularizations (MNVs) to identify potential biomarkers that could predict the required injection frequency throughout the disease course. **Methods:** In all patients, the initial diagnosis of nAMD was confirmed using optic coherence tomography (OCT), fluorescein angiography, and OCT angiography (OCTA). MNVs detected using OCTA were subjected to quantitative vascular analysis of their area, total vascular length (sumL), fractal dimension (FD), and flow density. These results were then correlated with the number of intravitreal anti-VEGF treatments administered during the first 3 years of treatment. Additionally, the relationship between the parameters and visual acuity progression was analyzed. **Results:** A total of 68 treatment-naïve eyes were included in the study, comprising 31 eyes with type 1 MNV, 19 eyes with type 2 MNV, and 18 eyes with type 3 MNV. The average MNV area at baseline was 1.11 mm^2^ ± 1.18 mm^2^, the mean total vascular length was 12.95 mm ± 14.24 mm, the mean fractal dimension was 1.26 ± 0.14, and the mean flow density was 41.19 ± 5.87. On average, patients in our cohort received 19.8 ± 8.5 intravitreal injections (IVIs). A significant correlation was found between the number of administered IVIs in the first 3 treatment years and the MNV area (*p* < 0.005), sumL (*p* < 0.005), and FD (*p* < 0.05), while no correlation was found with flow density. Additionally, there was no significant association between MNV type and treatment requirements, nor between MNV vascular architecture and visual acuity progression. **Conclusions:** The results suggest that the specific vascular structure of untreated MNV may serve as a predictor of long-term treatment demand. With the emergence of new drug classes and advancements in imaging techniques, these parameters could offer valuable insights for forecasting treatment requirements.

## 1. Background

Age-related macular degeneration (AMD) is the leading cause of visual impairment in the elderly population in Western countries [1]. The advanced stages of AMD are classified into two forms: geographic atrophy (GA), characterized by the progressive loss of photoreceptor and retinal pigment epithelium (RPE) cells, and neovascular AMD (nAMD), which involves the rapid growth of macular neovascularization (MNV) [2]. These neovascularizations lead to fluid accumulation within or beneath the retina or below the RPE [3]. This can also lead to the development of photoreceptor and RPE cell atrophy, even in the presence of MNV. According to the recently updated nomenclature, MNVs are categorized into three types based on the correlation between OCT imaging and histopathological findings: type 1 MNV involves choroidal vessels penetrating Bruch’s membrane (BM) and lying beneath the RPE; type 2 MNV grows from the choroid through the RPE into the subretinal space; and type 3 MNV originates in the retina and extends toward the RPE [4]. Diagnosis typically combines ophthalmoscopy and central visual acuity testing, with the gold standard for imaging being spectral-domain optical coherence tomography (SD-OCT) complemented by fluorescein angiography (FA) [5]. However, FA has the disadvantage of being an invasive procedure that requires the intravenous administration of fluorescein to patients. Additionally, it only provides an en face view of the retina up to the choroid, without the ability to selectively visualize individual layers. Furthermore, due to leakage, the vascular structure of the MNV appears blurred, making it difficult to assess accurately.

Optical coherence tomography angiography (OCTA), a modality developed from optical coherence tomography (OCT), detects the movement of blood cells and renders this motion as a grayscale image in contrast to the adjacent static tissues [6,7]. The retinal and choroidal blood vessels are thus depicted noninvasively [8]. A further advantage of OCTA over FA is that the vessels can be viewed segment-wise and in three dimensions, with the vascular structure portrayed in detail [9]. This permits objective mathematical description of MNV and shows previously unseen differences in patterns of disease [10,11,12]. We have also observed that individual differences in MNV vascular architecture influence the clinical course, including pathological morphological changes, particularly the distribution of intraretinal and subretinal fluid [12]. Additionally, it affects the therapeutic response after the initial upload phase with anti-VEGF therapy and disease progression during the first year of treatment [13,14]. Patients with AMD typically require long-term treatment, often spanning several years, involving frequent intravitreal anti-VEGF injections (IVI) and regular follow-up visits. However, there is significant variability among patients in terms of the required injection frequency and the progression of visual outcomes over the course of treatment, making it challenging to predict the individual disease trajectory.

The aim of this study was to explore whether specific characteristics of the vascular architecture of MNV, as identified through OCTA and MNV quantification at the time of diagnosis, could provide insights into the required frequency of anti-VEGF injections during the initial years of treatment. Additionally, an analysis was conducted to examine whether the vascular parameters of MNV have an impact on patients’ visual outcomes.

## 2. Methods

This study was conducted in adherence to the tenets of the Declaration of Helsinki and was approved by the ethics committee of the Westphalia–Lippe Medical Association and the University of Münster.

**Study Population:** This was a retrospective analysis of consecutive patients with newly diagnosed nAMD. Using an automated search function, patients were selected from patients’ digital medical records who had received an initial diagnosis of nAMD at our center, had undergone OCTA imaging before the first treatment, and had been followed up for examinations and treatments at our center over a period of three years. General information such as age, sex, best-corrected visual acuity, number of required anti-VEGF treatments, and history of other ocular diseases was recorded for all patients. In all participants, nAMD was initially diagnosed by means of FA and SD-OCT (Spectralis© HRA + OCT, Heidelberg Engineering, Heidelberg, Germany) with clinical examination. Each diagnosis was confirmed by a senior grader at the Reading Center of M^3^ Macula Monitor Münster, based on the distinctive features of the different MNV types observed through multimodal imaging [4]. All patients were treated with Ranibizumab, Aflibercept, or Bevacizumab using an activity-guided PRN regimen (IVAN scheme with triple injections). The data on the frequency of intravitreally administered anti-VEGF agents and visual acuity were extracted from the patients’ digital medical records.

**OCTA Image Analysis:** In addition to OCT and FA, all patients underwent OCTA imaging at the time of diagnosis using the swept-source OCTA device PLEX^®^ Elite 9000 (Carl Zeiss Meditec, Dublin, CA, USA). This device operates at a wavelength of 1060 nm with a scanning rate of 100,000 A-scans per second, capturing two consecutive B-scans of 500 A-scans each within a 6×6 mm field of view. The automatic artifact suppression feature was enabled to enhance the visualization and quantification of MNV, providing more accurate and reliable imaging results [15]. For the analysis of MNV, we selected segmentation from the outer retina to the choriocapillaris (ORCC). Due to the frequent occurrence of incorrect segmentation caused by morphological changes in the retina, all B-scans displaying the MNV were thoroughly reviewed twice. Whenever necessary, the segmentation lines were carefully adjusted and manually corrected to ensure accuracy.

**Figure 1:**
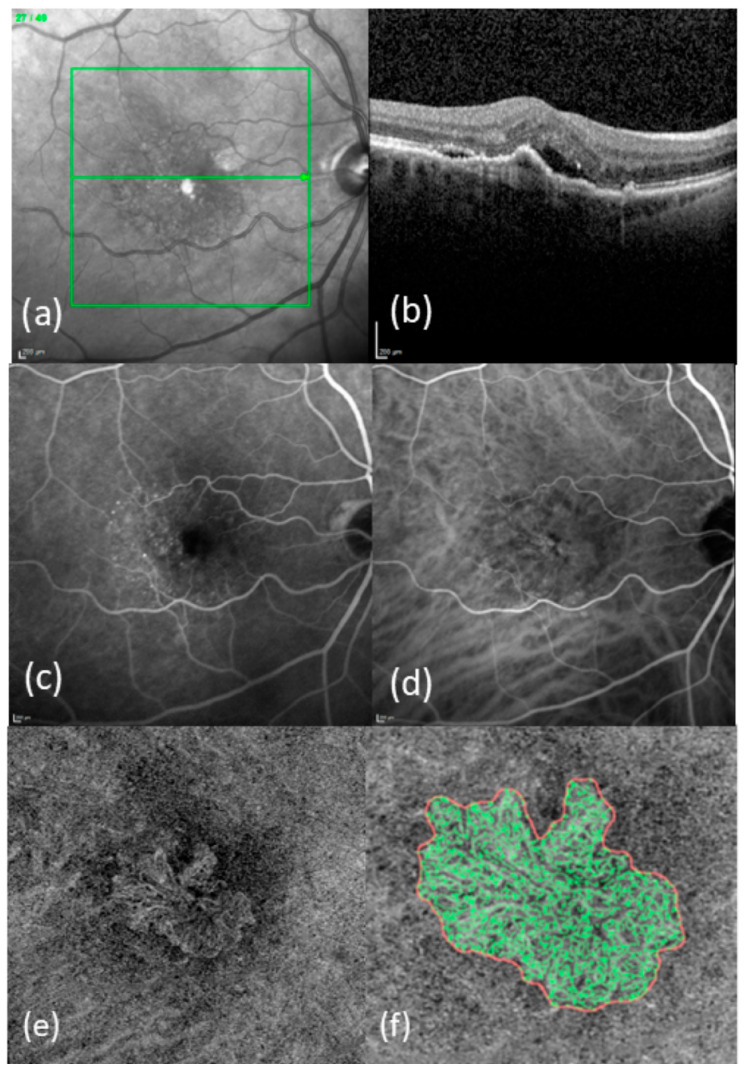
Typical images from patients with newly diagnosed nAMD: (**a**) IR-image with OCT line; (**b**) SD-OCT image; (**c**) FA; (**d**) ICGA; (**e**) en-face OCTA in ORCC segmentation; (**f**) enlarged and binarized MNV (red line = boundary of the MNV, green lines = vessels of the MNV).

In the exported OCTA images, the MNV was delineated in the en face view using the program Fiji (National Institute of Mental Health, Bethesda, MD, USA), and the MNVs were isolated from the rest of the image and saved for further analysis. The vascular network of the MNV was extracted with the aid of MatLab (Mathworks, Version R2021b; Natick, MA, USA). Skeletonization of the vascular network was achieved through the multiscale calculation of the gradient field in the en face OCTA image. This technique, described in detail elsewhere [16,17], depicts both wide and very narrow vascular segments as unbroken midlines. The following four vascular parameters were chosen for morphological characterization of the MNV structure: area, flow density, fractal dimension (FD), and total vascular length (sumL). The area represents the two-dimensional extent of the MNV in the en face view, while the sumL refers to the total length obtained by summing all individual MNV vessel segments. FD is a mathematical measure of the overall complexity of a structure and has previously been identified as a significant parameter of changes during anti-VEGF therapy [18,19]. Flow density is a measure of the proportion to which an MNV is vascularized and can be shown by all commercially available OCTA devices; it can thus be analyzed with no need for complex image processing. These data were correlated with the number of administered IVIs during the first three years of treatment. Figure 1 illustrates multimodal imaging (FA, SD-OCT, and OCTA) in the right eye of a patient with newly diagnosed nAMD, together with the images resulting from binarization and skeletonization of the data.

**Exclusion Criteria:** Patients with insufficient OCTA image quality (quality score < 6) were excluded, as were those with any retinal pathology other than nAMD. Additionally, patients with coexisting retinal diseases, such as diabetic retinopathy or retinal vascular occlusions, were excluded, as well as those in whom OCTA failed to detect MNV or where imaging was compromised by artifacts. Furthermore, patients were excluded if the MNV extended beyond the 6 × 6 mm imaging window, preventing a complete analysis.

**Statistics:** The data were evaluated with the statistics software R (version 4.3.2). The level of significance was set at 5% for all analyses. In this paper, we present the mean ± standard deviation to describe the distribution of a numeric variable and the contingencies to describe categorical data. For correlation tests, we chose weighted regression models to measure the correlation of the independent variables (IVs) and dependent variables (DVs): generalized weighted linear regression for at least interval-scaled DVs or generalized weighted logistic regression for dichotomic DVs, respectively. If a corresponding baseline variable was given, this was added to the model as a confounder. Weights were introduced by the geometric mean weights of the involved variables to reduce the influence of far outliers by the 10% and 90% percentiles ($p_{10}$ and $p_{90}$) as follows:

$$w\left(x\right)=\exp-0.5\cdot {\left(\frac{\max\left\{0,t_{low}-x,x-t_{high}\right\}}{0.5\cdot (t_{high}-t_{low})}\right)}^{2}where$$

$$t_{low}=p_{10}-1.5\cdot \left(p_{90}-p_{10}\right)\;and\;t_{high}=p_{90}+1.5\cdot \left(p_{90}-p_{10}\right)$$

## 3. Results

After applying our quality criteria, inclusion and exclusion criteria, and considering the 3-year follow-up period, 68 eyes (19 = male; 49 = female) from 64 patients were included in our final analysis. The patients’ mean age was 77.9 ± 7.8 years, and their best-corrected visual acuity at the initial baseline was 0.58 ± 0.33 logMAR and 0.54 ± 0.40 logMAR at the three-year follow-up.

The classification of MNV according to multimodal imaging revealed type 1 MNV in 31 patients, type 2 MNV in 19 patients, and type 3 MNV in 18 patients in our cohort.

No significant correlations were observed between the different MNV types and the number of IVIs (*p* = 0.49) administered during the first three years of treatment, nor with the visual acuity outcomes (*p* = 0.34).

In the morphological analyses of the MNV at baseline, the mean MNV area was 1.106 mm^2^ ± 1.181 mm^2^, the mean sumL was 12.95 mm ± 14.24 mm, the mean FD was 1.263 ± 0.145, and the mean flow density was 41.19 ± 5.87. The patients in our cohort received 19.8 ± 8.5 IVIs within the first three years after the initial diagnosis.

Three of the analyzed MNV vascular parameters present at the time of initial diagnosis demonstrated a significant correlation with a higher number of intravitreal anti-VEGF injections during the first three years of treatment. Specifically, larger MNV area (*p* < 0.005), longer total vascular length (sumL) (*p* < 0.005), and higher fractal dimension (FD) (*p* < 0.05) were associated with a greater number of injections during this period. In contrast, flow density did not show a significant impact on the number of required treatments (*p* = 0.207).

None of the MNV vascular parameters showed an influence on the visual acuity development of the patients (area: *p* = 0.475; sumL: *p* = 0.588; FD: *p* = 0.213; flow density: *p* = 0.773).

## 4. Discussion

So far, predictive factors regarding nAMD progression and treatment need have primarily been identified using SD-OCT. Bogunovic et al. identified the presence of subretinal fluid as the strongest predictor for injection requirement within the first 23 months [20]. In a study focusing on the visual acuity progression of nAMD patients within the first year, Waldstein et al. identified subretinal fluid, posterior vitreous detachment, and intraretinal fluid [21] as relevant predictive factors in SD-OCT. The predictive parameters of the considered studies, such as intraretinal and subretinal fluid, mainly concern the retinal exudative fluid distributions and are only secondary effects of the vascular changes in MNV. Thus, they provide only indirect insights into these vascular changes.

OCT-A has demonstrated high sensitivity and specificity in detecting MNV and enables a detailed examination of the vascular morphology of MNV in nAMD [4,22,23]. This allows for the classification of various MNV vascular morphologies, providing new insights into differences in the disease. It also facilitates the identification of characteristic vascular features [12,24]. It has also been shown that MNV vascular morphology changes rapidly with anti-VEGF therapy—especially small capillary vessels, which become undetectable just a few days after treatment. This suggests that the MNV vascular configuration may also contain prognostic markers for treatment response [25,26]. The potential of AI-driven analysis of OCTA data has been demonstrated by several studies. For instance, Heinke et al. achieved an accuracy of up to 78% in distinguishing wet from dry AMD using OCTA data across different OCTA devices [27]. Jin et al. further highlighted the superiority of AI algorithms in analyzing OCTA data compared to OCT data when differentiating between active and inactive disease stages [28]. Additionally, Schranz et al. showed in a deep learning-based OCTA analysis that the vascularized portion of an MNV at diagnosis serves as a predictive marker for the presence of intraretinal fluid after 12 weeks [29]. These findings underscore the growing relevance of AI-assisted OCTA analysis in the assessment and management of neovascular AMD.

Reliable predictive markers for treatment response in this disease are still lacking. In previous studies, we have described the influence of MNV architecture on different retinal morphologies—such as fluid distribution and retinal pigment epithelium elevation [12]—the therapeutic response to initial anti-VEGF therapy [13], and visual acuity progression as well as therapy requirements during the first treatment year [14]. Therefore, the aim of this exploratory study was to develop mathematical characterizations of the vascular morphology of treatment-naïve MNV in nAMD, which would allow for a prognosis of therapy requirements during the first three years of treatment.

The area and sumL of the MNV are size parameters and show in our cohort that a larger MNV at the time of initial diagnosis leads to an increased therapy requirement (treatment need) in the initial treatment years. This is consistent with the findings of Schranz et al., who observed significantly more therapy-resistant intraretinal fluid in larger MNV membranes [29]. This indicates that larger MNV membranes exhibit higher activity levels during exudation. Additionally, other OCT-A studies have shown that the perfused area of the MNV in OCT-A initially decreases with anti-VEGF therapy, but with renewed activity increase, an enlargement of the perfused area is observed, indicating that even with prolonged anti-VEGF therapy, perfusion of the MNV remains [17,30,31,32]. However, we were not able to demonstrate this correlation between MNV size parameters and increased therapy requirement in a previous study with a 1-year follow-up [14].

In our cohort, the FD of the MNV is also higher, and the vascular architecture is therefore more complex in patients with a higher treatment requirement. This aligns with our observations of MNV after the initial upload phase with anti-VEGF therapy, where signs of activity were more frequently present in cases with more complex MNV vascular architecture compared to simpler vascular structures [13]. Al-Sheikh et al. also demonstrated a correlation between high MNV FD and the degree of activity [18].

The flow density describes the percentage of pixels with a flow signal relative to the total number of pixels in the measured area of the MNV. In the present cohort, we did not observe any correlation between flow density and therapy requirement or visual acuity progression during the first 3 treatment years. However, the results may be influenced by the fact that “no flow” does not mean an absence of vessels, as only erythrocytes moving within a detectable speed range in the vessels are detected and ultimately represented as vessels [33]. Also, flow may not be detected if it moves toward or away from the detector. All of these factors lead to the conclusion that the flow value alone does not allow for a definitive statement about the vascular configuration of the MNV.

When analyzing whether type 1, 2, or 3 MNV exhibited different therapy requirements within the first 3 years, we could not identify any significant correlations. These categories were established based on the correlation between OCT images and histopathological examinations. However, they do not seem to play a significant role in the clinical course. Bogunovic et al. also demonstrated in a machine learning-based prediction model for the disease progression of nAMD that the MNV type had the lowest relevance among 50 parameters [20]. This highlights the importance of OCTA, as it provides added value through the detailed visualization of vascular architecture. However, processing vascular architecture remains labor-intensive and cannot currently be performed with commercial devices but instead requires external software for analysis.

The study is subject to potential selection bias due to its retrospective analysis. Several limitations must be considered: On the one hand, in addition to the described MNV characteristics, retinal changes are relevant as additional influencing factors for visual acuity prognosis and the number of injections. On the other hand, another central limitation is the resolution capacity of OCT-A imaging, which is particularly relevant when investigating fine vascular structures, such as capillaries. The collected parameters of the MNV in OCTA, such as area, total vessel length, FD, and flow density, thus only represent the perfused portion of the MNV in a given two-dimensional segmentation. Additionally, the MNV had to be manually marked, which always introduces a certain degree of subjectivity in delineating the MNV. Automated vessel skeletonization is also subject to the limitation that vessels may be incorrectly identified. Therefore, in future studies with larger cohorts, the use of artificial intelligence methods to correlate as many retinal and MNV parameters as possible is expected to enable an even better analysis of predictive factors for visual acuity progression and treatment intensity.

Deep learning is currently transforming scientific knowledge at an unprecedented pace, as vast amounts of data can be extracted and analyzed with remarkable efficiency. The field of medical imaging is particularly well suited for these advancements, and numerous studies have already employed AI algorithms to enhance understanding of neovascular age-related macular degeneration (nAMD). Since OCT imaging has been in clinical use for a longer period, it has become a standard diagnostic tool in routine examinations of nAMD patients and is also widely utilized in clinical trials for drug approval. As a result, extensive datasets are available, making OCT an ideal modality for AI-driven analyses. However, it would be highly desirable for OCTA to also be integrated into routine diagnostics and regulatory studies to expand the available data pool for AI evaluations.

Given that macular neovascularization (MNV) is the primary target of pharmacological therapy in nAMD, the expectation is that OCTA-based analyses could identify more precise biomarkers than those derived solely from secondary morphological changes in the retina detected using OCT. By incorporating OCTA into AI-driven research, there is significant potential to improve disease monitoring and treatment strategies in nAMD.

In summary, the MNV vascular architecture at initial diagnosis already provides indications of the number of necessary IVIs, enabling better patient education and preparation for therapy. The OCTA vascular parameters collected in this study had no influence on long-term visual acuity. The conventional classification into MNV types 1, 2, and 3 had no influence on either treatment frequency or visual acuity progression during the first three years.

## Data Availability

The data presented in this study are available on request from the corresponding author. The data are not publicly available due to privacy or ethical restrictions.
